# Improving reproductive outcomes in frozen embryo transfer over fresh embryo transfer in women with endometrioma: A historical cohort study

**DOI:** 10.18502/ijrm.v22i11.17819

**Published:** 2025-01-10

**Authors:** Parisa Pirooznia, Mehri Mashayekhi, Firouzeh Ghaffari, Nadia Jahangiri, Zahra Zolfaghari, Firoozeh Ahmadi, Fateme Hasani, Nima Narimani

**Affiliations:** ^1^Department of Endocrinology and Female Infertility, Reproductive Biomedicine Research Center, Royan Institute for Reproductive Biomedicine, ACECR, Tehran, Iran.; ^2^Department of Basic and Population Based Studies in NCD, Reproductive Epidemiology Research Center, Royan Institute, ACECR, Tehran, Iran.; ^3^Department of Reproductive Imaging, Reproductive Biomedicine Research Center, Royan Institute for Reproductive Biomedicine, ACECR, Tehran, Iran.; ^4^Department of Embryology, Reproductive Biomedicine Research Center, Royan Institute for Reproductive Biomedicine, ACECR, Tehran, Iran.; ^5^Hasheminejad Kidney Centre, School of Medicine, Iran University of Medical Sciences (IUMS), Tehran, Iran.

**Keywords:** Endometrioma, Embryo transfer, Assisted reproductive technique, Treatment outcome, Pregnancy outcome.

## Abstract

**Background:**

Endometrioma, a common manifestation of endometriosis, often indicates the severity of the disease. In vitro fertilization and embryo transfer (ET) are key therapeutic strategies for infertility associated with endometriosis. However, the optimal type of ET (frozen or fresh) and its impact on pregnancy success rates remain debated, with limited studies available.

**Objective:**

This historical cohort study aimed to compare fertility and neonatal outcomes, focusing on live birth rate (LBR), clinical pregnancy, and implantation rates in women with endometrioma-associated infertility, between fresh and frozen embryo transfer (FET).

**Materials and Methods:**

In this historical cohort study, the medical records (files) of 289 women diagnosed with endometrioma-related infertility, who underwent in vitro fertilization/intracytoplasmic sperm injection treatment at Royan Institute, Tehran, Iran between March 2016–2021 were reviewed. Ultimately, 200 files that met the established criteria were selected for review. The extracted data was then compared between groups: FET (n = 121) and fresh ET (n = 79).

**Results:**

No significant differences were observed between the groups in terms of demographic characteristics and endometrioma size. The only significant difference in fertility outcomes was the LBR, which was 36.4% for the FET group compared to 22.8% for the fresh ET group (p = 0.04). No significant differences were observed in neonatal outcomes between the groups. Overall, our study suggests that FET may lead to higher LBRs in women diagnosed with endometrioma.

**Conclusion:**

Our study suggests that FET may lead to higher LBRs in women diagnosed with endometrioma.

## 1. Introduction

Endometriosis is a chronic gynecological estrogen-dependent condition characterized by the ectopic proliferation of endometrial tissue outside the uterine cavity, predominantly on the ovaries, pelvic peritoneum, and rectovaginal septum (1, 2). A significant association between endometriosis and infertility is suggested by the fact that approximately half of the infertile individuals have endometriosis, and 30–50% of those diagnosed with endometriosis experience infertility (3, 4).

Ovarian endometrioma, a prevalent form of endometriosis, is typically identified by the presence of ovarian cysts filled with a chocolate-like fluid. The incidence of ovarian endometrioma among individuals diagnosed with endometriosis ranges from 17–44%, indicating that it is considerably more common than pelvic endometriosis (5–7).

The occurrence of endometriomas is often indicative of the severity of endometriosis. It has been observed that 50% of individuals with deep infiltrating endometriosis also present with concurrent endometriomas (8, 9). Endometriomas are particularly common in individuals undergoing planned cycles of in vitro fertilization (IVF), intracytoplasmic sperm injection (ICSI), or frozen embryo transfer (FET), with prevalence ranging from approximately 20–40% (7, 10).

The mechanisms through which endometriomas may impair fertility remain a subject of ongoing speculation. A meta-analysis has indicated a decrease in the number of oocytes and the clinical pregnancy rate (CPR) among individuals with endometriosis undergoing IVF treatment, compared to a control group (11, 12). On the other hand, it is postulated that endometrial factors may adversely affect implantation, thereby serving as a primary contributor to reduced fertility in individuals diagnosed with endometriosis (12).

Recently, an innovative approach known as the “freeze-all” has been attributed to increased pregnancy rates and a decrease in the risks of preterm birth, low birth weight, and perinatal mortality compared to the prevalent practice of fresh embryo transfer (ET) (13). It is plausible to suggest that the heightened stimulation associated with fertility treatments could potentially exacerbate disease progression and negatively impact endometrial receptivity and subsequent implantation. Various strategies have been proposed to prepare the endometrium in FET cycles. These include natural cycle, modified natural cycle with human chorionic gonadotropin, hormone replacement therapy (HRT) with or without gonadotropin-releasing hormone (GnRH) agonist, and induction of ovulation stimulation cycles (14–17). However, none of these methods have demonstrated clear superiority over others in terms of fertility outcomes (16, 17). Nevertheless, the optimal protocol for endometrial preparation in individuals with endometriosis continues to be a contentious topic within the scientific community (16).

At present, the medical community is engaged in a robust debate concerning the most effective strategy for addressing infertility linked to endometriosis. Consequently, the primary objective of this study is to compare the impacts of frozen and fresh ET on pregnancy and neonatal outcomes in individuals affected by endometrioma.

This study aimed to compare fertility and neonatal outcomes, focusing on live birth rate (LBR), clinical pregnancy, and implantation rate (IR) in women with endometrioma-associated infertility, between fresh and FET.

## 2. Materials and Methods

### Study design and subjects

This historical cohort study analyzed data of 200 infertile women aged between 18 and 39 yr with endometrioma, selected from 289 medical records (files) of women treated at the Royan Institute in Tehran, Iran, between March 2016 and 2021. The comparison was conducted between 2 groups: 121 files in the FET group and 79 files in the fresh ET group. The diagnosis of endometrioma was confirmed through ultrasound (transvaginal sonography).

In this study, the files included the data of women who had at least 2 good/excellent quality embryos (cleavage stage: 3-day embryos) and had undergone their first ET cycle with their own oocytes. Files that met any of the following criteria were excluded from the study: age ≥ 40 yr, preimplantation genetic diagnosis, uterine anomalies, severe male factor infertility (sperm count < 5 million/mL or azoospermia), history of endometriosis surgery, recurrent pregnancy loss, and anti-Müllerian hormone (AMH) levels < 1.1 ng/mL.

### Ovarian stimulation cycle

The type of ovarian stimulation protocol was chosen based on factors such as the individual's ovarian reserve, age, body mass index (BMI), and the physician's discretion (18). The standard protocols of stimulation included antagonist or GnRH agonist protocols (19, 20). In brief, following ovarian stimulation, oocyte retrieval was performed 34–36 hr after triggering (21), and fertilization was evaluated 16–18 hr after ICSI, with the presence of 2 pronuclei indicating successful fertilization (22). The generated embryos were assigned a grade of A or B, indicative of good to excellent quality, based on the cleavage stage, fragmentation pattern, and morphological characteristics of the embryos (23, 24).

### FET

The protocols for FET included 2 types: “GnRH agonist + HRT” and “ultralong” (25, 26). Once an endometrial thickness of ≥ 7 mm was confirmed, progesterone suppositories were administered, and 3-day embryos were transferred on the 4 ${\;}^{\text{th}}$ day of progesterone administration. In all groups, serum β -HCG levels were assessed 2 wk post-ET, and following a positive result, the same dosage of progesterone was continued for 12 wk.

### Outcomes

The primary outcome of this study was the LBR. Secondary outcomes included CPR, miscarriage rate (MR), and IR. LBR was calculated by dividing the number of women with at least one live birth by the number of women who underwent ET. CPR was defined as the number of pregnancies with fetal heart activity observed in the ultrasound examination at week 6 of pregnancy. MR was defined as the proportion of pregnancies that ended in loss before the 20 ${\;}^{\text{th}}$ wk of gestation. IR was calculated by dividing the number of gestational sacs by the number of embryos transferred.

Cases that resulted in loss before the 6 ${\;}^{\text{th}}$ wk of gestation, including blighted ovum and biochemical pregnancy, were excluded from the results due to their frequent association with fetal genetic abnormalities rather than defects in implantation. Gestational age was determined from the day of ET. Preterm labor is defined as childbirth occurring before the 37 ${\;}^{\text{th}}$ wk of gestation. Low birth weight and very low birth weight were characterized by a birth weight of < 2500 and 1500 gr, respectively. Macrosomia was defined as a birth weight exceeding 4000 gr.

### Ethical Considerations

The study protocol received approval from the Institutional Review Board and Ethics Committee of the Royan Institute, Tehran, Iran ensuring that all procedures adhered to ethical guidelines (Code: IR.ACECR.ROYAN.REC.1402.055). Additionally, data confidentiality was strictly maintained to protect personal privacy and proprietary information from unauthorized access and disclosure.

### Statistical Analysis 

Continuous and categorical variables were represented as mean (standard deviation), median (MD), interquartile range (IQR), and number (%), respectively. The normal distribution of variables was confirmed using the Kolmogorov-Smirnov test. The mean of the variables, as per the groups under study, was examined using an independent *t* test. In instances of non-normality, the Mann-Whitney U test was applied. The Chi-square test (Fisher's exact) was utilized to assess the relationship between categorical variables. Logistic regressions were executed to ascertain the independent relationships between individual characteristics and LBRs. Statistical significance was set as a p-value ≤ 0.05. Data analysis was conducted using SPSS 24.0 statistical software.

## 3. Results 

**Figure 1:**
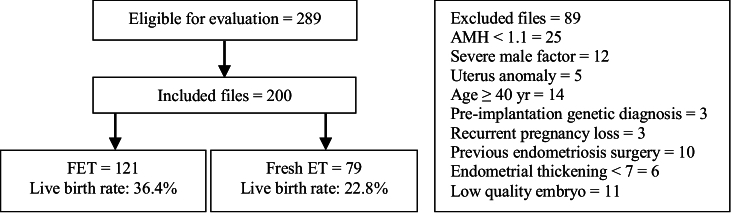
Flow chart of the cohort screening in the study. AMH: Anti-Müllerian hormone, ET: Embryo transfer, FET: Frozen embryo transfer.

After reviewing 289 files, 89 were found not meeting the inclusion criteria. Ultimately, 200 women with endometrioma were compared based on the available information in 2 groups: FET (n = 121) and fresh ET (n = 79) (Figure 1).

### Demographic characteristics

As delineated in table I, the baseline characteristics of the participants were compared between groups. The comparative analysis of mean age, BMI, duration of infertility, and type of infertility did not yield statistically significant differences between the 2 groups. Similarly, the mean baseline follicle-stimulating hormone and AMH levels did not exhibit statistically significant disparities between groups. The size of endometrioma and tumor marker levels were similar between groups. The prevalence of hydrosalpinx and the rate of corrective surgery for hydrosalpinx were also comparable between groups (Table I).

### Cycle characteristics

The protocol of ovarian stimulation cycles, gonadotropin dose, endometrial thickness, difficulty of ET, and number of transferred embryos were similar between groups. Despite similar AMH levels, the number of oocytes retrieved, and the number of metaphase II (MII) oocytes were statistically significantly different. A univariate logistic regression analysis was conducted to assess the impact of the number of oocytes and MII oocytes on the LBR; however, no significant effect was observed (OR: 1.03, CI: 0.943–1.109, p = 0.58). The method of endometrial preparation also did not influence the LBR (OR: 0.866, CI: 0.411–1.820, p = 0.70) (Table II).

### Fertility outcomes

The outcomes were compared between groups of participants undergoing fresh and frozen transfers, encompassing the rates of clinical pregnancy, miscarriage, live birth, and implantation. Notably, the LBR was found to be significantly different. It is also worth mentioning that no instances of ectopic pregnancy were observed in either of the 2 groups (Table III). Additionally, table IV presents a comparison of pregnancy outcomes between the 2 types of freezing protocols.

### Neonatal outcomes

Table V presents the neonatal outcomes. The 2 groups did not differ significantly in terms of type of delivery, gestational age, preterm pregnancy, multiple pregnancy, gender of infants, mean birth weight, low birth weight, and very low birth weight. In summary, the 2 groups had similar rates of neonatal outcomes.

### Analysis of variables influencing live birth outcomes

The analysis of variables influencing live birth outcomes is presented in table VI. This table provides a comprehensive overview of the factors affecting LBRs, including demographic, clinical, and treatment-related variables.

**Table 1:** Comparison of demographic characteristics between fresh and frozen ET groups

**Variables**		**Fresh ET group (n = 79)**	**FET group (n = 121)**	**P-value**
Age (yr)*		32.26 ± 3.583 29 (28–32.5)	31.71 ± 4.140 32 (30–35)	0.35^c^
BMI (Kg/m^2^)*		25.02 ± 3.601 24.38 (22.03–27.34)	24.23 ± 3.491 24.31 (22.58–26.31)	0.26^c^
Infertility duration (yr)*		3.592 ± 2.790 1 (1–7)	3.596 ± 2.544 3 (1–5)	0.793^d^
Cause of infertility**				
	**Endometriosis**	68 (86.0)	108 (89.2)	
	**Male factor and endometriosis**	11 (14)	13 (10.8)	0.499^a^
Type of infertility**				
	**Primary**	67 (84.8)	105 (86.8)	
	**Secondary**	12 (15.2)	16 (13.2)	0.695^a^
**Simultaneous hydrosalpinx**		23 (29.1)	37 (30.5)		0.825^a^
**Corrective surgeries for hydrosalpinx**		7 (8.86)	8 (6.61)	0.555^a^
Basal hormone levels on day 3*				
	**FSH (IU/mL)**	6.245 ± 2.925 4.3 (3.95–5.8)	6.181 ± 2.480 6.9 (5–9.155)	0.976^d^
	**AMH (ng/mL)**	2.320 ± 1.074 2.4 (1.65–3.45)	2.550 ± 1.170 2 (1.15–3.42)	0.242^d^
	**ROMA**	6.090 ± 3.056 4.95 (3.095–6.995)	5.066 ± 2.761 4.29 (2.5–7.43)	0.12^c^
	HE4 increased**	0 (0)	1 (1.06)	0.435^b^
Left-size endometrioma**				
	** < 3 cm**	36 (70.5)	62 (68.9)	
	** ≥ 3 cm**	15 (29.5)	28 (31.1)	0.833^a^
Right-size endometrioma**				
	** < 3 cm**	31 (59.6)	45 (51.1)	
	** ≥ 3 cm**	21 (40.4)	43 (48.9)	0.331^a^
*Data presented as Mean ± SD, median (interquartile range). **Data presented as n (%). a: Chi-square test, b: Fisher's exact, c: Independent sample *t* test, d: Mann-Whitney. AMH: Anti-Mullerian hormone, BMI: Body mass index, ET: Embryo transfer, FET: Frozen embryo transfer, FSH: Follicle-stimulating hormone, HE4: Human epididymis protein 4, ROMA: Risk of ovarian malignancy algorithm				

**Table 2:** Comparison of cycle characteristics between fresh and frozen ET groups

**Variables**		**Fresh ET group (n = 79)**	**FET group (n = 121)**	**95% CI**	**P-value**
Protocol of ovarian stimulation*					
	**Long**	58 (73.4)	85 (70.2)	-	
	**Antagonist**	21 (26.6)	36 (29.8)	-	0.749^a^
Type of FET protocol*					
	**GnRH agonist + HRT**	-	55 (45.5)	-	
	**Ultralong**	-	66 (54.5)	-	-
Gonadotropin dose**		2305 ± 967.8 1575 (1312–2137)	2363 ± 1065 2475 (1950–3150)	(-352, 237.5)		0.696^d^
Endometrial thickness (mm)**		9.980 ± 1.833 9 (8–12.75)	9.967 ± 1.545 10 (8.95–10.75)	(-346, 231.7)	0.877^d^
Difficulty of ET*					
	**Easy**	78 (98.7)	117 (96.7)	-	
	**Difficult**	1 (1.3)	4 (3.3)	-	0.366^b^
Total no of ET**		2.050 ± 0.677 2 (1.5–2)	2.165 ± 0.521 2 (2–2.5)	(-0.282, 0.053)		0.086^c^
No of oocytes**		6.784 ± 3.152 7 (5.5–11.5)	10.47 ± 5.803 11 (7–13)	(-5.105, -2.283)	< 0.001^d^
No of MII**		5.632 ± 2.592 6 (5.5–6.5)	8.034 ± 3.997 7 (5.5–11)	(-4.963, -2.425)	< 0.001^d^
*Data presented as n (%), **Data presented as Mean ± SD, median (interquartile range). a: Chi-square test, b: Fisher's exact, c: Independent sample *t* test, d: Mann-Whitney. ET: Embryo transfer, FET: Frozen embryo transfer, GnRH: Gonadotropin-releasing hormone, HRT: Hormone replacement therapy, MII: Metaphase II					

**Table 3:** Comparison of fertility outcomes between fresh and frozen ET groups

**Variables**	**Fresh ET group (n = 79)**	**FET group (n = 121)**	**P-value**
Pregnancy test after ET*	25 (31.6)	53 (43.8)	0.085^a^
Implantation/ET**	0.6 ± 0.24 0.5 (0.5–0.75)	0.64 ± 0.26 0.5 (0.5–1)	0.542^d^
Clinical pregnancy/ET cycle*	23 (29.1)	49 (40.5)	0.101^b^
Miscarriage/ ET cycle*	5 (6.3)	5 (4.1)	0.520^b^
Live birth/ET cycle*	18 (22.8)	44 (36.4)	0.042^a^
*Data presented as n (%). **Data presented as Mean ± SD, median (interquartile range). a: Chi-square test, b: Fisher's exact, d: Mann-Whitney. ET: Embryo transfer, FET: Frozen embryo transfer			

**Table 4:** Comparison of pregnancy outcomes between the 2 types of freezing protocols

**Variables**	**GnRH + HRT**	**Ultralong**	**P-value**
**Clinical pregnancy/ET cycle**	28 (50.9)	25 (37.8)	0.709
**Live birth/ET cycle**	21 (38.1)	23 (34.8)	0.198
ET: Embryo transfer, GnRH: Gonadotropin-releasing hormone, HRT: Hormone replacement therapy			

**Table 5:** Comparison of the neonatal outcomes between fresh and frozen ET groups

**Variables**		**Fresh ET group (n = 79)**	**FET group (n = 121)**	**P-value**
Gestational age (wk)*		36.14 ± 3.472 38 (34.5–38.5)	36.20 ± 2.923 37 (37–38)	0.942^d^
Type of birth**				
	**Term**	15 (83.33)	34 (77.28)	
	**Preterm**	3 (16.67)	10 (22.72)	0.739^b^
Method of delivery**				
	**C/S**	16 (88.9)	43 (97.72)	
	**NVD**	2 (11.1)	1 (2.28)	0.232^b^
**Multiple pregnancies**		5 (27.8)	11 (26.8)		0.588^b^
Type of pregnancy**				
	**Singleton**	13 (72.28)	33 (75)	
	**Twins**	5 (27.72)	10 (22.73)	
	**Triplets**	0 (0)	1 (2.27)	0.757^b^
Gender (singletons)**				
	**Female**	8 (61.62)	18 (54.55)		
	**Male**	5 (38.38)	15 (45.45)	0.749^a^
Gender (twins)**				
	**Female**	8 (80)	10 (50)	
	**Male**	2 (20)	10 (50)	0.235^b^
Gender (triplets)**				
	**Female**	0 (0)	3 (100)	
	**Male**	0 (0)	0 (0)	—
Weight (gr)*				
	**Singleton**	2846 ± 0.554	2939 ± 0.348	0.525^d^
	**Twins**	2400 ± 0.843	2100 ± 0.718	0.124^d^
Weight (singletons)**				
	**Low birth weight**	3 (23.0)	3 (9.09)	
	**Normal**	9 (69.21)	29 (87.88)	
	**Macrosomia**	1 (7.69)	1 (3.03)	0.323^b^
Weight (twins)**				
	**Very low birth weight**	2 (20)	4 (20)		
	**Low birth weight**	2 (20)	10 (50)	
	**Normal**	6 (60)	6 (30)	0.223^b^
**Pregnancy complications (pre-eclampsia)**		0 (0)	1 (0.82)		0.605^b^
*Data presented as Mean ± SD. **Data presented as n (%). a: Chi-square test, b: Fisher's exact, d: Mann-Whitney. C/S: Cesarian section, ET: Embryo transfer, FET: Frozen embryo transfer, NVD: Normal vaginal delivery				

**Table 6:** Univariate logistic regression analysis of the potential factors to the live birth

**Variables**		**OR**	**95% CI**	**P-value**
**Type of transfer**				
	**Fresh**	Reference group		
	**Freeze**	1.937	(1.017, 3.683)	0.044**
Age (yr)*		0.922	(0.852, 0.997)	0.042**
BMI(kg/m^2^)*		0.984	(0.892, 1.085)	0.744
Duration infertility (yr)*		0.944	(0.833, 1.069)	0.367
**Left size endometrioma**		0.968	(0.435, 2.148)	0.936
**Right size endometrioma**		0.656	(0.317, 1.357)	0.256
**Corrective surgeries**		1.123	(0.367, 3.434)	0.839
**Cause of infertility**		1.400	(0.527, 3.718)	0.500
**Endometrial thickness**		0.963	(0.801, 1.155)	0.683
**FSH**		0.927	(0.822, 1.044)	0.212
**AMH**		0.943	(0.700, 1.268)	0.697
**HE4**		0.000	(0)	1.000
**ROMA**		0.952	(0.824, 1.100)	0.508
**Number of embryos**		0.960	(0.808, 1.139)	0.640
**Simultaneous hydrosalpinx**		0.935	(0.484, 1.804)	0.841
**Type of FET protocol**		0.866	(0.411, 1.820)	0.704
**Trigger**				
	**Ovitrelle**	Reference group		
	**GnRH agonist**	0.908	(0.266, 3.088)	0.877
	**HCG**	2.250	(0.518, 9.767)	0.279
**Number of oocytes**		1.047	(0.989, 1.108)	0.110
**Number of MII oocytes**		1.023	(0.943, 1.109)	0.584
**Gonadotrophin**		1.000	(0.999, 1.000)	0.671
**Obtained by logistic regression statistically significant level at 0.05. AMH: Anti-Müllerian hormone, BMI: Body mass index, FET: Frozen embryo transfer, FSH: Follicle-stimulating hormone, GnRH-agonist: Gonadotropin-releasing hormone, HCG: Human chorionic gonadotropin, HE4: Human epididymis protein 4, MII: Metaphase II, ROMA: Risk of ovarian malignancy algorithm, OR: Odds ratio				

## 4. Discussion 

Our data suggest that the application of FET yields superior outcomes in infertile women diagnosed with endometrioma. In this investigation, the LBR/ET cycle was significantly elevated in the group undergoing frozen transfer. Although not reaching statistical significance, the CPR/ET cycle was also higher in the FET cohort. The rate of neonatal outcomes was comparable in both groups. Univariate logistic regression analysis revealed that the strategy of frozen transfer exerted a significant influence on the LBR (OR: 1.937, CI: 1.017–3.683, p = 0.044).

The optimal approach for managing infertility associated with advanced endometriosis remains a topic of ongoing debate within the medical community. A pertinent question is whether the freeze-all strategy can maintain optimal endometrial receptivity in individuals with endometriosis, thereby altering fertility outcomes.

The eutopic endometrium of individuals with endometriosis exhibits substantial biochemical and ultrastructural deviations when compared to normal tissue. Crucial molecules implicated in implantation, including integrin αVβ3 and *HOXA10*, are diminished in individuals with endometriosis. Additionally, the expression of the pro-implantation cytokine, leukemia inhibitory factor, is reduced in women with endometriosis.

The surge in estrogen during ovulation stimulation cycles promotes increased cell proliferation and upregulation of estrogen receptors in the endometrium, culminating in a deficiency in decidualization, a process essential for successful implantation. Endometriosis further disrupts this process through differential gene expression in the endometrium, alterations in cellular physiology, and vascular abnormalities (27).

In a retrospective cohort study comparing 3763 fresh ET cycles and 3523 frozen FET cycles, 415 cases (5.7%) were identified as having endometriosis-related infertility. In the cohort with endometriosis undergoing FET, where endometrial preparation was performed with a GnRH agonist, fertility outcomes were superior in individuals with endometriosis compared to those without the condition. The authors postulate that the administration of a GnRH agonist, in conjunction with reduced ovarian estrogen levels, creates a more conducive environment for implantation in individuals with endometriosis who underwent FET. However, this study did not provide information regarding the quality of the transferred embryos and the staging of endometriosis (15).

The first study that specifically examined individuals with endometriosis (135 fresh ET cycles and 135 FET cycles) showed that LBR and CPR are significantly higher in the freeze cycle compared to their fresh counterparts (28). Unlike the aforementioned studies that included all 3 types of endometriosis (deep infiltrating endometriosis, superficial peritoneal lesions and ovarian endometrioma), our cohort only included individuals with endometrioma, which might indicate the positive effect of FET in more severe cases. Furthermore, while the former study did not provide information about the quality of the transferred embryos and the staging of endometriosis, the latter had differences in ovulation cycles, the average number of transferred embryos, and the staging of transferred embryos between the fresh and the FET groups.

A retrospective study specifically targeting individuals with endometrioma compared the outcomes following blastocyst ET in both frozen and fresh groups. CPR, LBR, and IR were notably higher in the frozen transfer group. Similar to our study, the probability of miscarriage and multiple pregnancies was reported to be equivalent in both groups. The authors propose that frozen blastocyst ET may potentially be associated with an increased likelihood of fertility in these individuals. However, this study, by considering a sperm count of < 1 × 10^6^ as a severe male factor, has inadvertently overlooked numerous cases attributed to severe male factors. Furthermore, it failed to provide any information regarding the quality of the transferred embryos and did not examine the neonatal outcomes of pregnancy (29).

Another investigation compared fertility outcomes between 2 groups of frozen and fresh ET (30). The participant population in each group included individuals with stage I, II, and III/IV endometriosis. The groups exhibited similarities in terms of fertilization rate, IR, CPR, and MR. The results about the initial fertility outcomes align with our study, although their investigation was primarily focused on the initial pregnancy outcomes and did not examine the LBR. Furthermore, a separate comparison between endometriosis groups (stage I/II vs. stage III/IV) was not conducted.

A recent meta-analysis and systematic review compared frozen and fresh ET among individuals with a history of endometriosis (7). In total, 6 studies of moderate quality were considered, encompassing 3010 women diagnosed with endometriosis who were seeking fertility treatment. The LBR in the FET group was significantly higher, while the CPR was reported to be identical in both groups. This study requires clinical trials for confirmation, but none have been conducted in this field yet. Moreover, the heterogeneity of the meta-analysis is influenced by variations in the phenotype and staging of endometriosis, ovulation induction, ET protocols, number of transferred embryos, and maternal age among the study population, which impedes subgroup analysis.

Hence, it appears that the endometrium poses the primary impediment to implantation in women afflicted with active endometriosis (31).

This study was undertaken to investigate fertility and neonatal outcomes following the mitigation of the impact of surplus estrogen in FET cycles. In this investigation, we endeavored to minimize confounding factors by considering the most prevalent endometriosis phenotype, endometrioma, and implementing stringent restrictions, such as excluding cases with a history of endometriosis surgery and poor responder cases based on Bologna criteria. In light of the findings from studies that have not discerned a difference between ETs at the blastocyst stage and the cleavage stage (23, 32–36), and intending to minimize technical errors related to embryo development to the blastocyst stage including variations in the formulation and brand of the embryo culture medium we elected to employ embryos at the cleavage stage.

### Strengths and limitations

This study is subject to some limitations: firstly, the data selection may be influenced by bias due to the study's retrospective design. Secondly, we did not account for possible confounding factors, such as genetic and lifestyle variables. Lastly, the mental effects of undergoing the ICSI program may negatively affect the fertility outcomes of couples in fresh ET cycles.

Our study also has several strengths: first, the study was conducted in a population with endometrioma, which reduced potential bias. Second, neonatal outcomes were assessed, which are rarely reported in previous studies. Third, by excluding individuals aged 40 and over, we minimized the confounding effect of advanced maternal age on fertility outcomes. Fourth, by excluding individuals with AMH < 1.1 ng/mL, we eliminated the low responder group, which was considered a confounding factor in previous studies (32–36).

## 5. Conclusion

Our research indicates that the utilization of frozen embryos in the transfer process may result in an increased probability of LBR in individuals with endometrioma. This finding could significantly influence clinical practices and decision-making processes in the management of infertility related to endometrioma. It is important to note that while these results are promising, further research is needed to confirm these findings and to explore the underlying mechanisms involved. Nevertheless, this study provides a valuable contribution to our understanding of endometrioma-related infertility and potential treatment strategies.

##  Data Availability

Data supporting the findings of this study is available upon reasonable request from the corresponding author and the first author of the article.

##  Author Contributions

Concept and design: P. Pirooznia, M. Mashayekhi, and F. Ghaffari. Acquisition, analysis, or interpretation of data: F. Ghaffari and N. Jahangiri. Drafting of the manuscript: P. Pirooznia and M. Mashayekhi. Statistical analysis: Z. Zolfaghari. Supervision: N. Narimani. Diagnosing endometrioma through sonography: F. Ahmadi. Evaluating the quality of embryos: F. Hasani. All scientific content, including the literature review, data analysis, statistical analysis, discussion, and conclusions, was independently developed by the authors.

##  Acknowledgments

This research was not financially supported by any organization. The authors utilized the Artificial Intelligence tool Microsoft Copilot-GPT4 to assist with language editing and improving the readability of the manuscript.

##  Conflict of Interest

The authors declare no conflict of interest.
